# Interaction of *MDM33* with mitochondrial inner membrane homeostasis pathways in yeast

**DOI:** 10.1038/srep18344

**Published:** 2015-12-16

**Authors:** Till Klecker, Megan Wemmer, Mathias Haag, Alfons Weig, Stefan Böckler, Thomas Langer, Jodi Nunnari, Benedikt Westermann

**Affiliations:** 1 Universität Bayreuth, Zellbiologie, 95440 Bayreuth, Germany; 2 University of California Davis, Department of Molecular and Cellular Biology, Davis, CA 95616, USA; 3 Universität zu Köln, Institut für Genetik, 50931 Köln, Germany; 4 Universität Bayreuth, DNA Analytik, 95440 Bayreuth, Germany

## Abstract

Membrane homeostasis affects mitochondrial dynamics, morphology, and function. Here we report genetic and proteomic data that reveal multiple interactions of Mdm33, a protein essential for normal mitochondrial structure, with components of phospholipid metabolism and mitochondrial inner membrane homeostasis. We screened for suppressors of *MDM33* overexpression-induced growth arrest and isolated binding partners by immunoprecipitation of cross-linked cell extracts. These approaches revealed genetic and proteomic interactions of Mdm33 with prohibitins, Phb1 and Phb2, which are key components of mitochondrial inner membrane homeostasis. Lipid profiling by mass spectrometry of mitochondria isolated from Mdm33-overexpressing cells revealed that high levels of Mdm33 affect the levels of phosphatidylethanolamine and cardiolipin, the two key inner membrane phospholipids. Furthermore, we show that cells lacking Mdm33 show strongly decreased mitochondrial fission activity indicating that Mdm33 is critical for mitochondrial membrane dynamics. Our data suggest that *MDM33* functionally interacts with components important for inner membrane homeostasis and thereby supports mitochondrial division.

Mitochondria play key roles in cellular energy metabolism, various additional biochemical pathways, developmental processes, apoptosis, and aging. These functions are intimately linked to the shape of the mitochondrial compartment. Mitochondria are highly dynamic organelles that constantly adapt their morphology to the requirements of cellular physiology by frequent fusion and fission^1^,^2^ and remodeling of their ultrastructure^3^. As many of the molecular components and cellular pathways have been conserved during evolution, mitochondrial structure and dynamics can be studied in baker’s yeast *Saccharomyces cerevisiae* as a model organism. Fusion and fission of mitochondria are mediated by large dynamin-related GTPases (DRPs). Fzo1 in yeast and the mitofusins, Mfn1 and Mfn2, in mammals are membrane-bound proteins that constitute the key components of the outer membrane fusion machinery. Mgm1 in yeast and Opa1 in mammals are intermembrane space proteins that mediate inner membrane fusion. Mitochondrial fission is mediated by Dnm1 in yeast and Drp1 in mammals, which are cytosolic proteins that are recruited to the mitochondrial surface by membrane-bound receptors and adaptor proteins where they assemble into helical structures that sever the mitochondrial outer membrane. It is currently unknown whether a separate machinery for inner membrane division exists, or whether both mitochondrial membranes are severed simultaneously by the Dnm1/Drp1 helices assembled on the outer membrane^1^,^2^.

Mitochondria also display a complex organization at the ultrastructural level. The mitochondrial inner membrane consists of two functionally and compositionally distinct subcompartments: cristae, which are inner membrane invaginations that accommodate the respiratory chain complexes, and the inner boundary membrane, which is closely apposed to the outer membrane. These subcompartments are connected by narrow, tubular cristae junctions^3^. Several distinct protein complexes shape the mitochondrial inner membrane in a manner that is interdependent with the lipid composition of the organelle, however, their exact modes of action are not fully understood. The inner membrane fusion DRP, Mgm1/Opa1, is thought to play an independent role in maintenance of inner membrane structure and organization^4^,^5^. ATP synthase dimerization and higher order assembly are also essential for cristae formation and stabilization^6^. More recently, a large complex of inner membrane associated proteins, termed mitochondrial contact site and cristae organizing system (MICOS), has been implicated as a master organizing system of the inner membrane, which is thought to function as both a blueprint for cristae placement and cristae stabilization^7^,^8^,^9^.

The lipid composition of mitochondria also plays a critical role in mitochondrial shape and ultrastructure. Mitochondria of yeast cells lacking enzymes involved in cardiolipin (CL) biogenesis exhibit altered mitochondrial morphology, including extremely elongated cristae sheets that sometimes form inner membrane septae or onion-like structures^10^,^11^,^12^. Similarly, mitochondrial cristae defects are observed in *Arabidopsis* leaf cells, human lymphoblasts, and mouse cardiomyocytes defective in cardiolipin biogenesis^13^,^14^,^15^. Furthermore, mitochondrial morphological and ultrastructural defects are observed in yeast and mammalian cells with reduced phosphatidylserine (PS) or phosphatidylethanolamine (PE) levels^16^,^17^.

The yeast protein, Mdm33, has been implicated in both mitochondrial dynamics and ultrastructure^18^. Mdm33 is an integral mitochondrial inner membrane protein with a majority of residues predicted to form coiled coil structures facing the matrix. Mutants lacking Mdm33 contain large, extended mitochondria frequently forming hollow spheres that enclose portions of the cytoplasm. Aberrant Δ*mdm33* mitochondria contain swollen parts filled with cristae and extended parts that are devoid of cristae. Overexpression of Mdm33 causes mitochondria to become fragmented and to contain septated or vesiculated inner membranes and leads to growth arrest^18^. However, it is not clear whether Mdm33 plays a primary role in the organization of the inner membrane, or inner membrane division, and whether these functions are interconnected. To reveal the role of Mdm33 in mitochondrial shape we systematically analyzed its genetic and proteomic interactions. Our results suggest that *MDM33* functions in a genetic network with genes important for inner membrane homeostasis and thereby supports mitochondrial division.

## Results

### ***MDM33*** interacts with genes involved in phospholipid metabolism and mitochondrial inner membrane homeostasis

**Figure 1:**
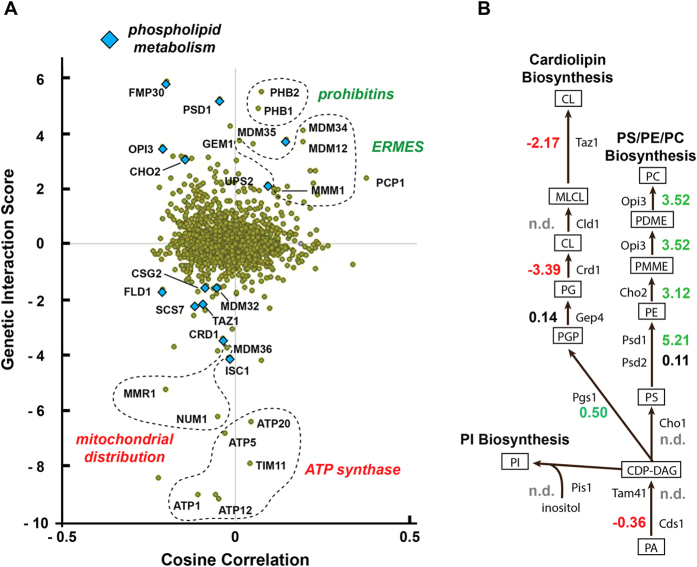
*MDM33* interacts with genes encoding components of phospholipid biosynthesis. (**A**) A genetic connection scatter plot of *MDM33* was generated using data from the MITO-MAP^8^. Each point in the scatter plot represents one gene. The x-axis represents the cosine correlation between the Δ*mdm33* interaction scores and the interaction scores obtained for the represented gene. The cosine correlation coefficients are generated by comparing the genetic interaction profiles of Δ*mdm33* to the profiles of all other 1,481 genes included in the MITO-MAP^8^. It represents a measure of the similarity of two genes’ genetic interactomes and serves as a measure of their functional similarity. The y-axis indicates interaction score between Δ*mdm33* and the represented gene. Several genes acting in phospholipid metabolism, mitochondrial inner membrane homeostasis (prohibitins), mitochondrial ER contacts (ERMES), mitochondrial distribution, and ATP synthase are highlighted. (**B**) Pathways of phospholipid biosynthesis. Positive (green), neutral (black), and negative (red) genetic interaction scores are indicated. CDP-DAG, cytidinediphosphate-diacylglycerol; CL, cardiolipin; MLCL, monolyso-cardiolipin; PA, phosphatidic acid; PC, phosphatidylcholine; PDME, phosphatidyldimethyl-ethanolamine; PE, phosphatidylethanolamine; PG, phosphatidylglycerol; PGP, phosphatidyl-glycerolphosphate; PI, phosphatidylinositol; PMME, phosphatidylmonomethyl-ethanolamine; PS, phosphatidylserine.

The MITO-MAP^8^ is based on 616,270 distinct pairwise genetic interactions of 1,482 genes and provides a comprehensive view of the connections between cellular pathways related to mitochondrial functions. Utilizing this resource we identified genes that positively or negatively interact with *MDM33* in double deletions (Fig. 1A; Table S1). Positive genetic interactions frequently occur between genes acting in a common pathway, whereas negative interactions point to compensatory pathways. Strikingly, *MDM33* shows strong positive interactions with the genes encoding enzymes required for synthesis of the major phospholipids, PS, PE, and phosphatidylcholine (PC), and negative interactions with genes required for CL biosynthesis (Fig. 1B). Furthermore, *MDM33* positively interacts with the prohibitin genes, *PHB1* and *PHB2*, which encode an inner membrane complex modulating mitochondrial phospholipid homeostasis^19^, and with genes encoding subunits of the ER mitochondria encounter structure (ERMES) that participates in the exchange of lipids between mitochondria and the ER^20^. Two clusters of negative genetic interactors comprise components of mitochondrial distribution and ATP synthase subunits (Fig. 1A). These data suggest roles of Mdm33 in phospholipid metabolism and mitochondrial membrane homeostasis, processes that are also critical for mitochondrial distribution and function.

To gain additional insight into Mdm33 function, we exploited the growth arrest phenotype of cells overexpressing *MDM33*^18^, reasoning that Mdm33 requires interaction partners to exert its function, and that these interaction partners will also be required to induce the growth arrest. Thus, deletion of a gene encoding an interaction partner required for Mdm33 function should relieve the overexpression-induced growth defect. Therefore, we identified putative suppressors by quantifying the abundance of deletion mutants in cell pools by a microarray-based approach (Fig. S1A,B).

**Figure 2:**
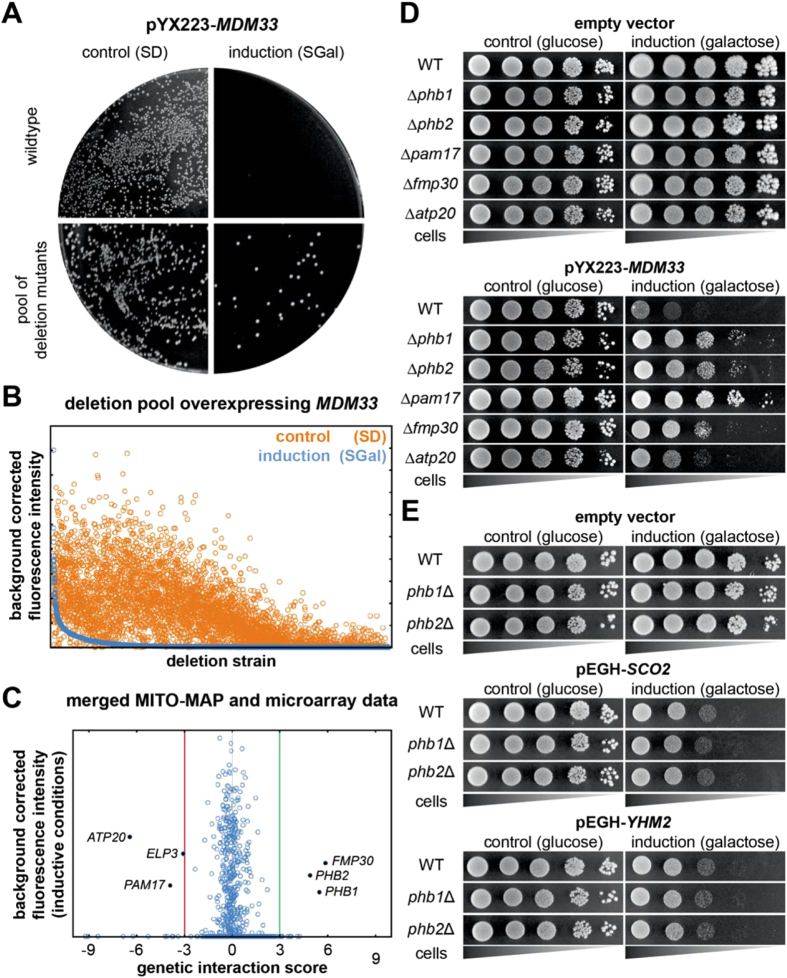
Genome-wide screen for suppressors of *MDM33* overexpression-induced growth arrest. (**A**) Wild type cells or pooled deletion strains were transformed with pYX223-*MDM33* (multicopy plasmid for expression of *MDM33* under control of the *GAL1/10* promoter) or the empty vector and plated on synthetic medium containing glucose (control (SD)) or galactose (induction (SGal)) as carbon source. Growth was observed after 3 days of incubation at 30 °C. (**B**) A pool containing the 4,987 strains of the *MAT*α haploid non-essential yeast deletion library was transformed with pYX223-*MDM33*, plated on synthetic medium containing glucose (control) or galactose (induction) as carbon source and strain abundance was quantified by microarray hybridization. Shown are normalized and background corrected microarray fluorescence signal values for the barcodes of each deletion strain. Strains are in descending order according to the fluorescence intensity under inducing condition (blue circles, arbitrary units). Fluorescence intensity under control condition is indicated by orange circles. (**C**) Scatter plot showing the genetic interaction score between *MDM33* and the represented gene according to the MITO-MAP (compare Fig. 1) and the normalized and background corrected microarray fluorescence signal taken from (**B**). (**D**) Strains were transformed with pYX223-*MDM33* for expression of *MDM33* under control of the *GAL1/10* promoter or the corresponding empty vector. 10-fold serial dilutions were spotted on synthetic complete medium containing glucose or galactose as carbon source and incubated at 30 °C for 2 (glucose) or 8 (galactose) days. In this and the following figures all samples that are shown in the same figure received an identical treatment in the same experiment. (**E**) Strains were transformed with an empty vector or a multicopy plasmid for overexpression of *SCO2* or *YHM2* from the *GAL1/10* promoter. 10-fold serial dilutions were spotted on synthetic complete medium containing glucose or galactose as carbon source. Growth was scored after incubation at 30 °C for 3 days.

To induce *MDM33* overexpression-dependent growth arrest we transformed a multicopy plasmid expressing *MDM33* from the strong, inducible *GAL1/10* promoter into a pool containing the 4,987 strains of the *MAT*α haploid non-essential yeast deletion library^21^. We expected that only deletion mutants that are unable to express *MDM33* properly or lack Mdm33 interaction partners would grow under inducing conditions. In a proof-of-principle experiment we plated a similar number of transformants of the isogenic wild type and the deletion mutant pool on repressing and inducing media. As expected, transformants of the wild type and pooled deletion mutants grew well on glucose-containing medium (SD) when *MDM33* overexpression was repressed. Wild type transformants ceased to grow on galactose-containing medium (SGal) when *MDM33* overexpression was induced. In contrast, some transformants of the deletion pool produced colonies under inducing conditions (Fig. 2A) suggesting that yeast cells are able to cope with high Mdm33 levels when critical interacting genes are lacking.

To screen for genes interacting with *MDM33*, we grew the transformed yeast deletion pool under repressing and inducing conditions on agar plates. As each strain of the yeast deletion library is marked with a unique molecular barcode sequence^21^ (Fig. S1A) microarray hybridization can be used to quantify deletion mutants in pools (Fig. S1B). We isolated genomic DNA from the pooled transformants and determined the abundance of the deletion mutants under inducing vs. repressing conditions in two independent experiments. After high-density oligonucleotide array hybridization, approximately 80% of the deletion mutants produced a good signal after growth on glucose-containing medium, but were not detectable after growth on galactose-containing medium (Fig. 2B). As expected, Δ*gal3* and Δ*gal4*, which lack factors important for induction of the *GAL1/10* promoter, grew well under inducing conditions (Fig. S1C). Several hundred mutants lacking proteins of various cellular functions produced a significant signal under inducing conditions and thus represent putative interaction partners of *MDM33* (Table S2).

To identify candidates that genetically interact with *MDM33* upon both overexpression and deletion we merged the results from the suppressor screen with the MITO-MAP (interaction score less than -3 or more than 3). Six genes showed high scores in both data sets (Fig. 2C). *ELP3* encodes a subunit of the RNA polymerase II holoenzyme and is responsible for transcriptional elongation^22^. This gene shows a very high number of genetic interactions and was excluded from further analysis as it likely interacts with *MDM33* in an indirect manner. The remaining five genes encode mitochondrial proteins, namely the two subunits of the prohibitin complex, Phb1 and Phb2^19^; a factor known to be associated with mitochondrial lipid metabolism, Fmp30^23^; subunit g of the ATP synthase, Atp20^24^; and a regulatory subunit of the mitochondrial protein import motor complex, Pam17^25^.

After transformation of the *MDM33*-overexpressing plasmid into deletion mutants, we confirmed that absence of Atp20, Fmp30, Pam17, Phb1, or Phb2 suppresses the growth defect of cells (Figs 2D and S2B). It has been observed that overexpression of several genes encoding mitochondrial inner membrane proteins is toxic in yeast^26^. To test whether our screen yielded suppressors specific for *MDM33* we examined the effect of overexpressing two non-related genes encoding mitochondrial inner membrane proteins, *SCO2* and *YHM2*, that have been reported to cause a growth arrest when expressed from the *GAL1/10* promoter^26^. Sco2 acts in the delivery of copper to the cytochrome c oxidase^27^, whereas Yhm2 catalyzes mitochondrial citrate/oxoglutarate antiport^28^. Deletion of the prohibitin genes did not affect the growth of *SCO2* or *YHM2* overexpressing strains (Fig. 2E). We conclude that the suppression of overexpression-induced growth defects in our suppressor mutant strains is specific for *MDM33*. In addition, we confirmed that deletions of prohibitin genes and *MDM33* display positive genetic interactions (Fig. S2A,B), as indicated by the MITO-MAP. Taken together, our genetic analysis suggests that Phb1, Phb2, Pam17, Fmp30, Atp20, and Mdm33 act in closely related cellular pathways. In the case of Pam17 we consider it possible that the Δ*pam17* deletion compromises the activity of the TIM23 protein import machinery and thereby reduces the levels of Mdm33 accumulating in mitochondria and renders the cells more resistant to *MDM33* overexpression.

Next, we asked whether the overexpression of prohibitins would cause a growth arrest, similar to *MDM33* overexpression. As Phb1 and Phb2 are functionally interdependent we overexpressed both proteins simultaneously from the same plasmid under control of the *GAL1/10* promoter^29^. This overexpression resulted in a slight growth defect (Fig. S2C). However, simultaneous overexpression of Phb1 and Phb2 together with Mdm33 was almost lethal (Fig. S2C). This result confirms the genetic interactions and points to a common, non-redundant role of Mdm33 and prohibitins in the same cellular pathway.

In addition to its role in ATP synthesis the ATP synthase plays an important role in mitochondrial cristae biogenesis^3^. We reasoned that defects in ATP synthesis, but not in cristae formation might be phenocopied by growth on oligomycin-containing medium, an inhibitor of the ATP synthase. Addition of 5 μg/ml oligomycin to the medium prevented growth of wild type cells on non-fermentable carbon sources, suggesting that ATP synthase activity is effectively blocked under these conditions (data not shown). However, on fermentable medium the addition of oligomycin had no effect on the growth of wild type or Δ*mdm33* cells (Fig. S2D), suggesting that the interactions with genes encoding ATP synthase subunits are not caused by defects in ATP synthesis. Intriguingly, Atp20 is an ATP synthase subunit mediating dimerization of the ATP synthase complex, a process crucial for cristae biogenesis^3^,^6^. The identification of this gene both in the MITO-MAP and in the *MDM33* overexpression screen suggests that the role of the ATP synthase in mitochondrial structure, rather than ATP synthesis, is important for the genetic interactions with *MDM33*.

### *Mdm33* is in proximity of Phb1, Phb2, and Atp20

To test whether the genetic interactions reflect physical proximity of the proteins in cells, we searched for Mdm33 interaction partners using a proteomic analysis. Specifically, we chemically cross-linked lysates of cells expressing a functional GFP-Mdm33 fusion using DSP and purified Mdm33 using anti-GFP antibodies. Interacting proteins were identified by mass spectrometry (LC-MS/MS). Analysis of total spectral counts indicated that the most robust interacting proteins were the prohibitins, which were detected proportionally to Mdm33 in purifications from cells expressing GFP-Mdm33 at endogenous levels from an allele integrated at the *MDM33* locus, a low-copy, or a multi-copy plasmid. In addition, Atp1 and Atp2, the alpha and beta subunits of the F_1_ sector of the ATP synthase, were found to interact with GFP-Mdm33 (Table 1). Thus, proteomic analysis suggests proximity between the Mdm33 complex, prohibitins, and the ATP synthase. These results are in good agreement with the genetic interactions of *MDM33* with *PHB1*, *PHB2*, and *ATP20*.

### Phospholipid homeostasis rather than fusion-fission dynamics is required for the formation of aberrant mitochondria in Δ*mdm33* cells

**Figure 3:**
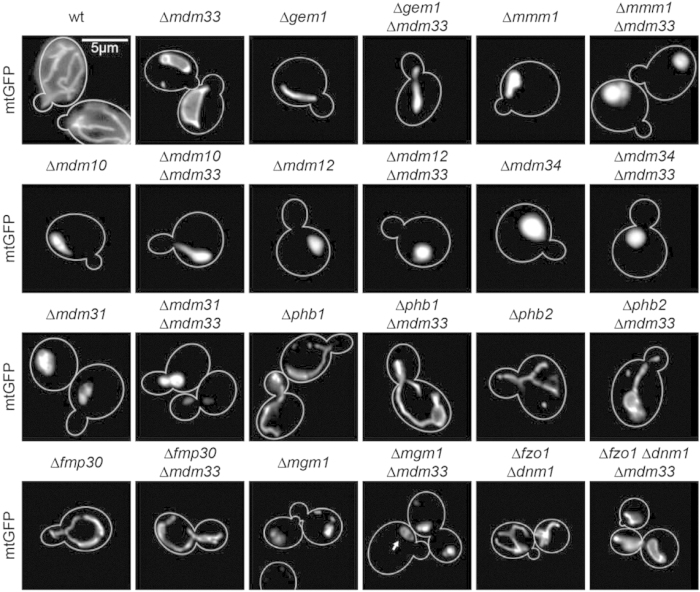
Formation of aberrant mitochondrial structures in Δ*mdm33* cells depends on components of inner membrane homeostasis, but not fusion and fission pathways. Cells expressing mitochondrial matrix targeted GFP (mtGFP) were grown to logarithmic growth phase in YPD and analyzed by fluorescence microscopy. Cell outlines are indicated by a white line. The arrow indicates smaller spherical mitochondria in Δ*mgm1* Δ*mdm33* double mutant cells. Bar, 5 μm. See also Table 2.

Cells lacking *MDM33* have a distinct mitochondrial morphological defect that includes formation of characteristic lariat-shaped structures and hollow spheres^18^. Therefore, we systematically examined mitochondrial morphology in cells with combined deletions of the *MDM33* gene and genes identified in our genetic and proteomic approaches and also genes encoding proteins directly involved in mitochondrial structure or dynamics (Table 2). Δ*mdm33* was found to be epistatic to most of the other deletion alleles, i.e. the Δ*mdm33* mitochondrial morphology phenotype was dominant, substantiating the central role of Mdm33 in mitochondrial morphogenesis. However, deletion of *FMP30*, *GEM1*, *MDM10*, *MDM12*, *MDM31*, *MDM34*, or *MMM1* resulted in a mitochondrial morphology phenotype that was epistatic to Δ*mdm33*, i.e. the distinct characteristic mitochondrial morphological defect of Δ*mdm33* was largely lost in these double mutants (Fig. 3). Δ*mdm33* Δ*phb1* and Δ*mdm33* Δ*phb2* double mutants showed an intermediate phenotype with largely tubular mitochondria and only few, small lariat-like structures (Fig. 3). Intriguingly, all of these genes are implicated in mitochondrial lipid metabolism: (i) Mdm10, Mdm12, Mdm34, and Mmm1 are core components of the ERMES complex that forms contact sites connecting the ER with mitochondria and participates in the import of lipids from the ER into mitochondria^20^,^30^,^31^. Gem1 is a regulator of ERMES^32^,^33^. (ii) Both Fmp30 and prohibitins are known to show strong genetic interactions with genes involved in mitochondrial CL and PE biosynthesis, and Fmp30 is required for maintenance of CL in the absence of mitochondrial PE synthesis^23^,^34^,^35^. (iii) Mdm31 is known to play an important role in CL biosynthesis in mitochondria, and overexpression of *MDM31* can partially compensate for the loss of ERMES^36^. The requirement of mitochondrial phospholipid biosynthesis factors for the manifestation of the characteristic Δ*mdm33* mitochondrial phenotype further supports a potential role of Mdm33 in this pathway.

In contrast, double mutants lacking the *MDM33* gene and genes encoding components of the mitochondrial fission machinery, *DNM1*, *FIS1*, or *MDV1*, resembled that of Δ*mdm33* single mutants. Lariat-shaped mitochondria characteristic for Δ*mdm33* could be found in double mutants lacking mitochondrial fusion factors Fzo1, Mdm30, or Mgm1. Mitochondria of these double mutants were more fragmented than that of Δ*mdm33* single mutants due to the fusion defect (Table 2). Triple mutants lacking Mdm33 and the outer membrane fusion and fission factors Fzo1 and Dnm1 appeared like the Δ*mdm33* single mutant (Fig. 3). Thus, mitochondrial fusion-fission dynamics is not required for formation of lariat-shaped mitochondria in cells lacking Mdm33. These results suggest that Mdm33 acts upstream of mitochondrial fusion and fission.

### Mdm33 affects phospholipid homeostasis in mitochondria

**Figure 4:**
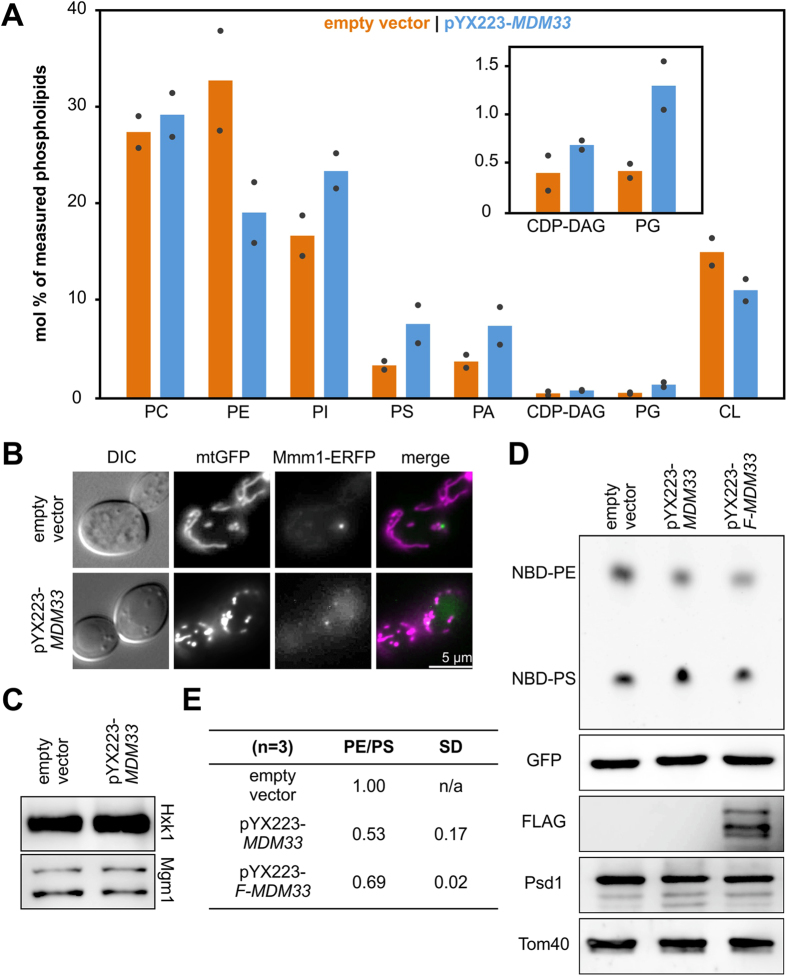
Mdm33 affects mitochondrial phospholipid homeostasis. (**A**) Phospholipidome of mitochondria isolated from cells transformed with pYX223-*MDM33* for expression of *MDM33* under control of the *GAL1/10* promoter (blue) or the corresponding empty vector (orange) and grown in synthetic complete medium containing galactose as carbon source. Phospholipid analysis was done by quantitative mass spectrometry. Bars represent the mean values of two independent mitochondrial preparations. The mean values of two technical replicates for each of the mitochondrial preparations are indicated by black dots. (**B**) Wild type cells expressing mitochondrial matrix targeted GFP (mtGFP), and ERFP-tagged Mmm1 (pRS316-MMM1-ERFP) were transformed with pYX223-*MDM33* or the empty vector, grown to the logarithmic growth phase in synthetic complete medium containing galactose as carbon source, and analyzed by fluorescence microscopy. Bar, 5 μm. (**C**) Western blot analysis of whole cell extracts of wild type cells carrying an empty vector or pYX223-*MDM33*. Cells were grown overnight in synthetic complete medium containing galactose as carbon source and diluted to logarithmic growth phase. Protein was extracted from the cells by boiling in sample buffer after alkaline treatment. (**D**) *In vitro* Psd1 activity assay. Mitochondria were isolated from wild type cells carrying an empty vector or overexpressing Mdm33 (pYX223-*MDM33*) or a FLAG-tagged version of Mdm33 (pYX223-*F-MDM33*) and incubated with liposomes containing NBD-PS for 30 minutes at 30 °C. Total lipids were isolated and separated by TLC. Shown is the NBD-fluorescence. The same samples were analyzed by Western blotting. (**E**) Quantifications are the ratio of the NBD-PE and NBD-PS signals normalized to the wild type-ratio. Shown are mean values and standard deviation obtained from three independent experiments.

**Figure 5:**
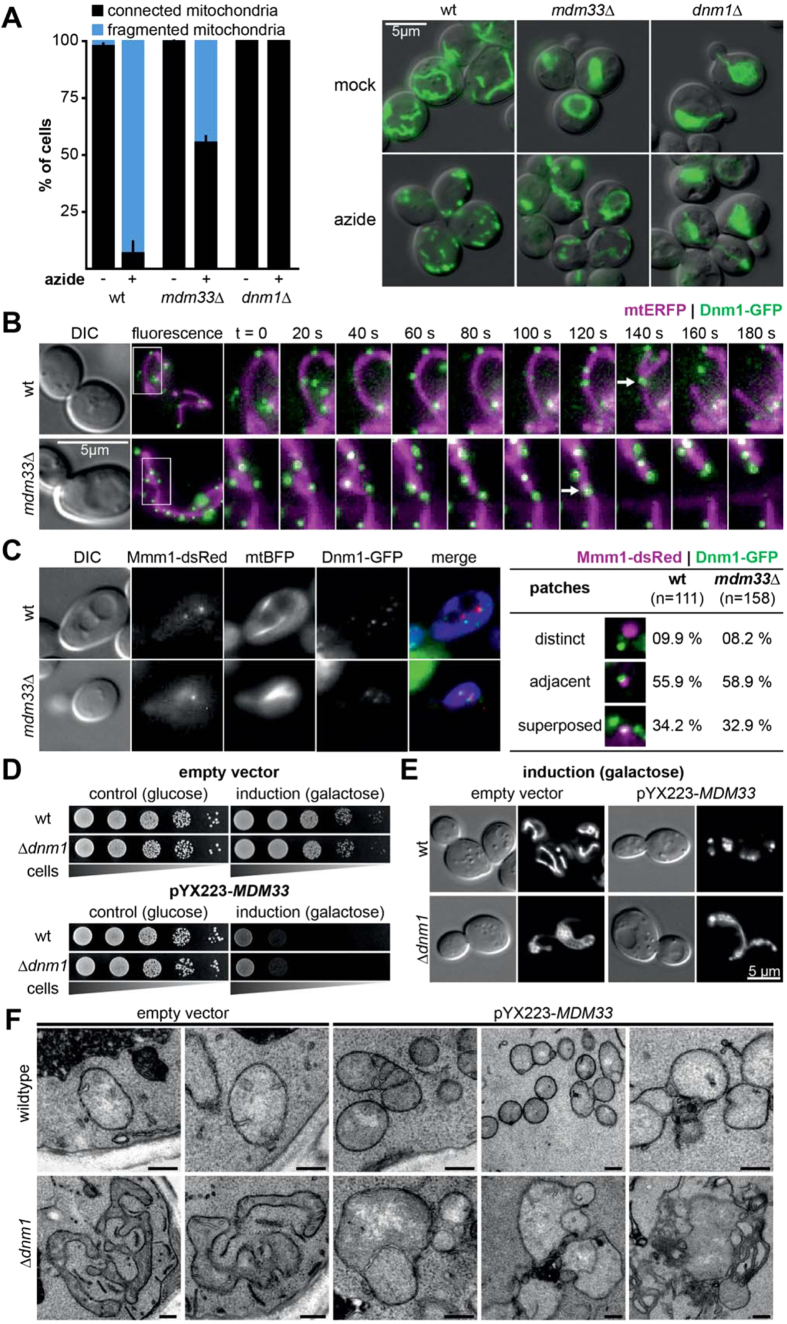
Mdm33 is required for mitochondrial fission. (**A**) Cells expressing mitochondrial matrix targeted GFP (mtGFP) were grown to logarithmic growth phase in YPD, either mock-treated or incubated for 40 min with 0.5 mM sodium azide, fixed in 3.7% formaldehyde, and analyzed by fluorescence microscopy. For each strain cells containing fragmented mitochondria were scored. Error bars indicate standard deviations of 3 independent experiments with 150 cells per experiment. Images are merges of DIC and GFP fluorescence. Bar, 5 μm. (**B**) Time lapse series of cells grown to logarithmic growth phase in synthetic complete medium and expressing mitochondrial matrix targeted ERFP (mtERFP) and Dnm1-GFP. Images represent maximum intensity projections of fluorescence image z stacks. Arrows highlight mitochondrial division events. Bar, 5 μm. (**C**) Cells expressing Dnm1-GFP, Mmm1-ERFP, and mitochondria targeted BFP (mtBFP) were grown to logarithmic growth phase in synthetic complete medium and analyzed by fluorescence microscopy. For both strains cells were scored for association of Mmm1-ERFP with Dnm1-GFP patches. Bar, 5 μm. (**D**) Strains were transformed with a multicopy plasmid for overexpression of *MDM33* from the inducible *GAL1/10* promoter (pYX223-*MDM33*) or an empty vector. 10-fold serial dilutions were spotted on synthetic complete medium containing glucose (repression of the *GAL* promoter) or galactose (induction of the *GAL* promoter) as carbon source and incubated at 30 °C for 2-4 days. (**E**) Control cells (empty vector) and cells overexpressing *MDM33* from the *GAL1/10* promoter (pYX223-*MDM33*) and mtGFP were grown overnight in synthetic complete medium containing galactose as carbon source, diluted to logarithmic growth phase and analyzed by fluorescence microscopy. Bar, 5 μm. (**F**) Electron micrographs of ultrathin sections of cells grown as in (**E**). Bars, 200 nm.

Our genetic and proteomic analyses suggested that *MDM33* participates in the organization of the inner membrane and mitochondrial lipid homeostasis. Therefore, we analyzed the phospholipid composition of mitochondria isolated from wild type, Δ*mdm33* cells, or *MDM33*-overexpressing cells by mass spectrometry. Although deletion of *MDM33* had no measurable effect on mitochondrial phospholipid composition, phospholipids that are synthesized in mitochondria, PE and CL, were strongly reduced in mitochondria from cells overexpressing *MDM33* (Fig. 4A), suggesting that Mdm33 affects mitochondrial phospholipid homeostasis. The combined deletion of genes required for production of CL and PE is synthetic lethal in yeast, indicating that CL and PE are partially redundant and that a sufficient level of either of them is required for cell survival^37^. Thus, disturbance of mitochondrial phospholipid biosynthesis upon overexpression of *MDM33* might contribute to the observed growth defect. Furthermore, it is known that changes in mitochondrial lipid composition cause septation of the mitochondrial inner membrane^11^. Therefore, we consider it likely that alterations of the PE and CL content in mitochondrial membranes contribute to the mitochondrial ultrastructure defect observed upon overexpression of *MDM33*^18^ (Fig. 5F).

PE biosynthesis occurs via multiple pathways^38^ (Fig. S3). In yeast the main pathway starts in the ER where PS is synthesized. PS is then transported to the mitochondrial inner membrane where the PS decarboxylase, Psd1, converts it to PE^39^,^40^. Contact sites of ER and mitochondria play a major role in the import of lipids into mitochondria^20^,^41^,^42^,^43^. Therefore, we checked whether the formation of ERMES, which was suggested to play a role in phospholipid exchange between both organelles^20^, is impaired by overexpression of *MDM33*. Impairment of ERMES is expected to change the localization of Mmm1, an ER-resident ERMES subunit, from patch-like assemblies to a diffuse ER signal^20^. Analysis of Mmm1-ERFP expressing strains by fluorescence microscopy revealed that ERMES localization is not affected by *MDM33* overexpression (Fig. 4B), suggesting that at least ERMES-dependent ER-mitochondria contact sites persist under these conditions.

Psd1 is required for processing of Mgm1^16^, which exists in two alternatively processed forms, both of which are required for mitochondrial inner membrane fusion^44^. As mitochondria are similarly fragmented in cells lacking Mgm1^45^ and in cells overexpressing *MDM33*^18^, we considered the possibility that altered lipid composition of mitochondria containing excess Mdm33 results in a defect in processing of Mgm1. However, Western blot analysis of total cell extracts revealed that this is not the case (Fig. 4C).

Next, we examined the conversion of PS to PE by addition of liposomes containing fluorescently labeled PS (NBD-PS) to isolated mitochondria and subsequent thin layer chromatography of mitochondrial lipids^46^. As expected, conversion of PS to PE was completely blocked when mitochondria were isolated from strains lacking phosphatidylserine decarboxylase activity (Fig. S4A). In contrast, mitochondria isolated from Δ*mdm33* cells showed wild type-like PS to PE conversion activity (Fig. S4B). Strikingly, mitochondria containing high Mdm33 levels showed only about 50-70% PS to PE conversion activity compared to the wild type (Fig. 4D,E). A Western blot analysis revealed that the protein level of Psd1 was not changed by *MDM33* overexpression (Fig. 4D). Taken together, our results suggest that the presence of excess Mdm33 has an impact on PE synthesis in mitochondria.

### Mdm33 contributes to mitochondrial division

Δ*mdm33* cells harbor large mitochondria that are often interconnected, and overexpression of *MDM33* results in mitochondrial fragmentation. These observations are suggestive of a role of Mdm33 in mitochondrial division^18^. To test a function of Mdm33 in this process more directly, we induced mitochondrial fragmentation in wild type, Δ*dnm1*, and Δ*mdm33* cells by treatment with sodium azide and observed mitochondrial morphology by fluorescence microscopy (Fig. 5A). Treatment of wild type cells led to rapid mitochondrial fragmentation, while Δ*dnm1* mitochondria remained interconnected, confirming that fragmentation is dependent on the mitochondrial fission machinery. In contrast, Δ*mdm33* mutants retained considerable fission activity. However, the number of cells with fragmented mitochondria was strongly reduced compared to the wild type suggesting that Mdm33 contributes to mitochondrial fission (Fig. 5A). Next, we analyzed cells expressing Dnm1-GFP by time-resolved live cell fluorescence microscopy. We could observe Dnm1-GFP-dependent matrix constriction and mitochondrial division in Δ*mdm33* cells (Fig. 5B). However, these events were restricted to a rather small tubular portion of the mitochondria and never occurred in the large ring-like structures. We conclude that Mdm33 contributes to efficient mitochondrial division, albeit it does not appear to constitute an essential component of the mitochondrial division machinery.

ER tubules wrap around mitochondria and mediate mitochondrial constriction prior to Dnm1 assembly^47^. This is spatially and functionally linked to ERMES, which tethers the ER and mitochondria^20^,^30^,^48^. The Miro GTPase Gem1 is required to disintegrate these contacts after the division event, thereby generating motile mitochondrial tips^48^. We asked whether the association of ERMES with sites of mitochondrial division might be disturbed in cells lacking Mdm33. We observed that the ER-resident ERMES subunit Mmm1 colocalizes with Dnm1-GFP in wild type and Δ*mdm33* cells, suggesting that this step of mitochondrial division does not require Mdm33 (Fig. 5C).

To test whether mitochondrial fragmentation and growth arrest upon *MDM33* overexpression depend on the outer membrane fission machinery we overexpressed *MDM33* in Δ*dnm1* strains. We observed a strong growth defect in cells lacking Dnm1, although we did not observe mitochondrial fragmentation (Fig. 5D,E). Instead we found parts of the mitochondrial network to be swollen. Electron microscopy revealed that overexpression of *MDM33* in the absence of Dnm1 causes swelling of the mitochondria and inner membrane septae formation (Fig. 5F). This indicates that mitochondrial fragmentation upon *MDM33* overexpression is Dnm1-dependent, whereas growth arrest and inner membrane remodeling are independent of Dnm1. Taken together, our results are consistent with the idea that Mdm33 is important to keep mitochondria in a fission-competent shape.

## Discussion

**Figure 6:**
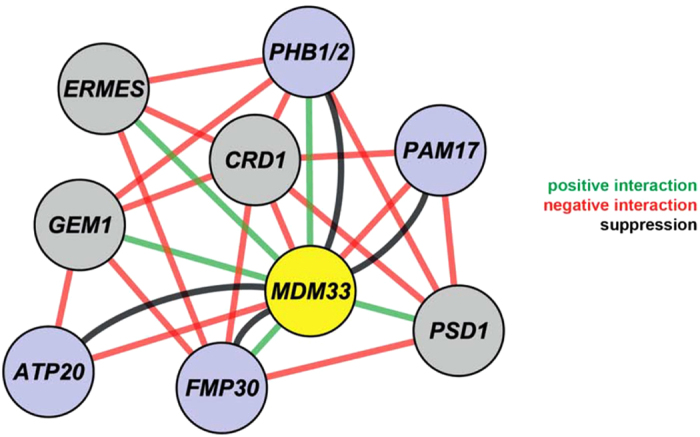
*MDM33* is part of a genetic network modulating mitochondrial phospholipid biosynthesis. Nodes in the genetic interaction network of *MDM33* represent deleted or overexpressed genes; connecting lines represent genetic interactions. The network is based on the scores obtained in the MITO-MAP (blue and grey) and in the suppressor screen (blue). Green and red lines indicate genetic interactions between deletion mutants (MITO-MAP); black lines indicate genetic interactions of *MDM33* overexpression with deletion mutants (suppressor screen). The genetic interaction network was created using the Cytoscape software^60^.

Here, we report several lines of evidence for an interaction of Mdm33 with molecular components and pathways of mitochondrial inner membrane homeostasis. The Δ*mdm33* deletion shows negative genetic interactions with genes required for synthesis of CL and positive genetic interactions with genes required for synthesis of the major phospholipids PS, PE, and PC, and with the prohibitin genes, *PHB1* and *PHB2*. The growth arrest upon *MDM33* overexpression is relieved by deletion of genes with a known role in mitochondrial lipid homeostasis, *PHB1*, *PHB2*, and *FMP30*. Taken together, our genetic analyses revealed a highly interconnected genetic interaction network also including ERMES, the ERMES regulatory subunit Gem1, and genes of the mitochondrial PE and CL biosynthesis pathways, namely *PSD1* and *CRD1* (Fig. 6). Furthermore, Mdm33 was found in crosslinks with Phb1 and Phb2. Thus, genetic data coupled with the proteomic analysis point to an involvement of Mdm33 in inner membrane phospholipid homeostasis.

Prohibitins are known modulators of mitochondrial inner membrane homeostasis and it is well established that they have an intimate functional relationship with the lipid composition of mitochondrial membranes, especially with the levels of PE and CL^19^. We previously proposed that ring-like prohibitin complexes might serve as membrane organizers modulating the distribution of PE and CL within the membrane^35^. Strikingly, Mdm33 overexpression lowers the levels of the same phospholipids that are also affected by prohibitins, namely PE and CL. An interaction of Mdm33 with prohibitins is further substantiated by three independent lines of evidence: a positive genetic interaction of prohibitin deletions with Δ*mdm33*, suppression of *MDM33* overexpression-induced growth arrest by prohibitin deletions, and a physical interaction of prohibitins with Mdm33 in cross-linked cell extracts. It is therefore conceivable that Mdm33 cooperates with prohibitins to modulate the proper organization of the inner membrane and the associated lipid biosynthetic pathways.

Accumulating evidence suggests that modulation of lipid composition is an important aspect of mitochondrial membrane fusion and fission^38^,^49^. For example, the biosynthesis of PE and CL is intimately linked to mitochondrial fusion in yeast^16^,^50^,^51^, CL plays a major role in mitochondrial division in *Arabidopsis thaliana*^52^, and CL stimulates the assembly of Mgm1/Opa1 and Drp1^51^,^53^,^54^. We show here that excess Mdm33 has an impact on the abundance of PE and CL. These are both non-bilayer forming lipids with small headgroups and therefore confer negative curvature to mitochondrial membranes, a feature that is particularly important during fusion or fission^55^. Thus, it is conceivable that Mdm33 activity affects the dynamic properties of the inner membrane.

Our previous characterization of the Δ*mdm33* mutant provided evidence for a role of Mdm33 in mitochondrial inner membrane division. Mutant cells have a unique phenotype with extremely extended and often ring-shaped giant mitochondria. We proposed that these aberrant organelles can form only in the absence of frequent division events^18^. Here, we show that deletion of the *MDM33* gene impedes azide-induced mitochondrial fragmentation. Furthermore, time-resolved fluorescence microscopy revealed that Dnm1 and ERMES-dependent mitochondrial fission events do occur in the absence of Mdm33, albeit at strongly reduced frequency. This indicates that Mdm33 plays an auxiliary role in mitochondrial fission, rather than being an essential part of the division machinery. Lariat-shaped mitochondria characteristic of mutants lacking *MDM33* are prevalent in double and triple mutants lacking fusion and fission factors. Thus, Δ*mdm33* is epistatic to the deletion of genes encoding core components of the fusion and fission machineries. Furthermore, Mdm33 overexpression-induced inner membrane septae form independently of Dnm1. These observations suggest that Mdm33 acts upstream of fusion and fission.

Taken together, our observations suggest that Mdm33 is in an ideal position to link mitochondrial inner membrane lipid homeostasis and structure to mitochondrial dynamics. We consider it possible that the activity of Mdm33 is required to prepare the inner membrane for division by local modulation of its phospholipid composition and biophysical properties. For example, Mdm33 might contribute to the formation of microdomains that are particularly enriched in PE and CL to generate curvature. Formation of these microdomains then renders the membrane competent for fission (i.e. fusion of the inner membrane from the matrix side). Future work will be required to test this idea and reveal whether Mdm33 is directly involved in this process.

The exact molecular role of Mdm33 remains unknown. The reduction of mitochondrial PE and CL levels upon Mdm33 overexpression points to an involvement in phospholipid metabolism. On the other hand, we did not detect an altered lipid composition of Δ*mdm33* mitochondria. Thus, it is possible that Mdm33 is not the limiting factor in wild type mitochondria, or that the activity of Mdm33 modulates the local distribution, rather than the absolute level, of mitochondrial phospholipids. It is equally well possible that Mdm33 indirectly affects membrane phospholipid homeostasis, e.g. by acting as a regulator of the prohibitin ring complex, or MICOS assembly, or ATP synthase dimerization. We are confident that the genetic and proteomic interaction network reported here will help to reveal the molecular function of Mdm33 in the future.

## Methods

### Yeast strain constructions

Plasmids and cloning procedures are described in the supplementary methods. Standard procedures were used for manipulation of yeast. Yeast deletion mutants were taken from the yeast deletion collection^21^. Double deletion mutants were constructed by mating and tetrad dissection. Yeast strains were isogenic to BY4741, BY4742, and BY4743^56^, if not indicated otherwise. For rapid generation of double mutants the synthetic genetic array (SGA) technology was used^57^. ORF replacement of *MDM33* was achieved by homologous recombination. The *URA3* marker used for this purpose was amplified using the plasmid pYES-mtGFP^58^ as template and oligonucleotides 5′-GAT CAT TGG GGT CTT TTT CGT TGT GAA ATT GTA ACG GGT GAA CTC AGT GAT TGA ATC TTA GAT CAC ACT GCC TTT G and 5′-TGT ATT TAT GAT TTT ATT ATG TAC AAG GAT AAA GGA TGA AAA AAA TGC ATG CGT GTT ACC GCT GTT GAG ATC CAG TTC.

### Microscopy

Epifluorescence microscopy was performed using an Axiophot or an Axioplan 2 microscope (Carl Zeiss Lichtmikroskopie, Göttingen, Germany) equipped with a Leica DCF360FX Camera with Leica LAF AF Version 2.2.1 Software (Leica Microsystems, Wetzlar, Germany) or an Evolution VF Mono Cooled monochrome camera (Intas, Göttingen, Germany) with Image ProPlus 5.0 and Scope Pro4.5 software (Media Cybernetics, Silver Spring, MD), respectively. For time-resolved live cell microscopy in Fig. 5B cells were observed with a Leica DMI 6000 wide field fluorescence microscope equipped with a Leica DFC360FX camera and Leica LAS AF Software Version 2.1.0. Image manipulations other than minor adjustments of brightness and contrast were not performed. For electron microscopy, cells were grown to log phase and prepared essentially as described^59^. Ultrathin 50 nm sections were poststained for 20 min with 2% uranyl acetate and for 3 min in lead citrate. Samples were examined in a Zeiss CEM 902 (Carl Zeiss, Oberkochen, Germany) transmission electron microscope operated at 80 kV. Micrographs were taken using a 1350 × 1050 pixel Erlangshen ES500W CCD camera (Gatan, Peasanton, CA) and Digital Micrograph software (version 1.70.16).

### Microarray design and hybridization, immunoprecipitation and LC MS/MS analysis

The rationale and procedure for microarray design and hybridization is outlined in the supplementary methods. Immunoprecipitation was performed basically as described previously^8^ with adjustments, as outlined in the supplementary methods.

### Lipid profiling by mass spectrometry and *in vitro* Psd1 activity assay

Mitochondria for lipid profiling and measurement of Psd1 activity were isolated from yeast cells by differential centrifugation and further purified by sucrose gradient centrifugation. Mass spectrometric phospholipid analysis of isolated mitochondria was performed as described^11^. The preparation of liposomes and the *in vitro* assay for Psd1 activity were performed essentially as described previously^46^. Lipids in stock solutions in chloroform were mixed at the desired molar ratio, and the solvent was evaporated under a flow of dry nitrogen. The dried lipids were hydrated in 800 μl of reaction buffer (300 mM sucrose, 150 mM KCl, 10 mM Tris-HCl, pH 7.5, 1 mM DTT) by repeated cycles of incubation at 30 °C, vortexing, and freeze-thawing. After 1 h hydration the liposomes were prepared by extruding 30 times at 30 °C using an Avanti Mini-Extruder with 0.2 μm polycarbonate membranes according to the manufacturer’s instructions. Liposomes were stored at 4 °C and used within 5 days.

For thin layer chromatography, phospholipids were extracted from mitochondria by vortexing in 500 μl of 2:1 chloroform/methanol for 15 min at room temperature using a Disruptor Genie (Scientific Industries, Bohemia, NY). 100 μl of water were added and the samples were vortexed for additional 5 min. The organic phase was separated by centrifugation at 400 x g for 5 min and dried under a constant flow of nitrogen. The samples were resuspended in 60 μl of chloroform and 20 μl of each sample was subjected to TLC analysis. Silica gel plates (Fluka Analytical, Sigma-Aldrich, St. Louis, MO) were developed with chloroform/methanol/acetone/water/acetic acid (50:10:20:5:15, vol/vol/vol/vol/vol). NBD fluorescence was imaged with an ImageQuant LAS 4000 gel documentation system (GE Healthcare Europe GmbH, Freiburg, Germany) using excitation and detection wavelengths for GFP.

## Additional Information

**Accession codes:** Microarray and sample annotation data were deposited at NCBI’s Gene Expression Omnibus (GEO) data repository under accession no. GSE49580.

**How to cite this article**: Klecker, T. *et al.* Interaction of *MDM33* with mitochondrial inner membrane homeostasis pathways in yeast. *Sci. Rep.* **5**, 18344; doi: 10.1038/srep18344 (2015).

## Supplementary Material

Supplementary Information

Supplementary Table 1

Supplementary Table 2

## Figures and Tables

**Table 1:** Mdm33 is in proximity of prohibitins and ATP synthase.

protein identified	total spectrum count (% coverage)		
	integrated GFP-Mdm33	low copy GFP-Mdm33	high copy GFP-Mdm33
Mdm33	203 (64%)	450 (76%)	512 (77%)
Phb1	8 (20%)	35 (56%)	45 (56%)
Phb2	13 (22%)	48 (49%)	65 (51%)
Atp1	9 (12%)	42 (24%)	54 (28%)
Atp2	9 (15%)	120 (60%)	74 (43%)

Immunoprecipitates of cross-linked GFP-Mdm33 were analyzed by mass spectrometry. The number of peptides and percent coverage is shown for each identified protein.

**Table 2:** Screen for genes that are required for the formation of aberrant mitochondrial structures in the Δ*mdm33* mutant.

Mutant	Interaction score	Mitochondrial phenotype
Δ*mdm33* Δ*atp12*	−9,2	Δ*mdm33*
Δ*mdm33* Δ*tpm1*	−9,03	Δ*mdm33*
Δ*mdm33* Δ*ptc1*	−8,42	Δ*mdm33*
Δ*mdm33* Δ*tim11*	−7,93	Δ*mdm33*
Δ*mdm33* Δ*atp5*	−6,82	Δ*mdm33*
Δ*mdm33* Δ*atp20*	−6,41	Δ*mdm33*
Δ*mdm33* Δ*num1*	−6,21	Δ*mdm33*
Δ*mdm33* Δ*mmr1*	−5,26	Δ*mdm33*
Δ*mdm33* Δ*yta12*	−4,21	Δ*mdm33*
Δ*mdm33* Δ*isc1*	−4,09	Δ*mdm33*
Δ*mdm33* Δ*pam17*	−3,87	Δ*mdm33*
Δ*mdm33* Δ*mdm36*	−3,76	Δ*mdm33*
Δ*mdm33* Δ*pep7*	−3,69	Δ*mdm33*
Δ*mdm33* Δ*crd1*	−3,39	Δ*mdm33*
Δ*mdm33* Δ*cap1*	−2,31	Δ*mdm33*
Δ*mdm33* Δ*taz1*	−2,17	Δ*mdm33*
Δ*mdm33* Δ*mic19*	−1,52	Δ*mdm33*
Δ*mdm33* Δ*mic60*	−1,26	Δ*mdm33*
Δ*mdm33* Δ*afg3*	−1,24	Δ*mdm33*
Δ*mdm33* Δ*mic12*	−1,03	Δ*mdm33*
Δ*mdm33* Δ*mdm32*	−0,54	Δ*mdm33*
Δ*mdm33* Δ*caf4*	−0,43	Δ*mdm33*
Δ*mdm33* Δ*dnm1*	−0,37	Δ*mdm33*
Δ*mdm33* Δ*ypt11*	−0,28	Δ*mdm33*
Δ*mdm33* Δ*mdv1*	−0,15	Δ*mdm33*
Δ*mdm33* Δ*ups1*	−0,06	Δ*mdm33*
Δ*mdm33* Δ*mgm101*	0	Δ*mdm33*
Δ*mdm33* Δ*mic26*	0	Δ*mdm33*
Δ*mdm33* Δ*fis1*	0,1	Δ*mdm33*
Δ*mdm33* Δ*mdm30*	0,35	Δ*mdm33*, fragmented
Δ*mdm33* Δ*mgm1*	0,38	Δ*mdm33*, fragmented
Δ*mdm33* Δ*mip1*	0,4	Δ*mdm33*
Δ*mdm33* Δ*mdm38*	0,71	Δ*mdm33*
Δ*mdm33* Δ*mdm10*	1,27	Δ***mdm10***
Δ*mdm33* Δ*ugo1*	1,42	Δ***ugo1***
Δ*mdm33* Δ*fzo1*	1,78	Δ*mdm33*, fragmented
Δ*mdm33* Δ*yme1*	1,92	Δ***yme1***
Δ*mdm33* Δ*mmm1*	1,99	Δ***mmm1***
Δ*mdm33* Δ*sur4*	2,03	Δ*mdm33*
Δ*mdm33* Δ*swi4*	2,08	Δ*mdm33*
Δ*mdm33* Δ*gep5*	2,1	Δ*mdm33*
Δ*mdm33* Δ*ctk1*	2,11	Δ*mdm33*
Δ*mdm33* Δ*snf1*	2,11	Δ*mdm33*
Δ*mdm33* Δ*erg3*	2,16	Δ*mdm33*
Δ*mdm33* Δ*ups2*	2,16	Δ*mdm33*
Δ*mdm33* Δ*slg1*	2,17	Δ*mdm33*
Δ*mdm33* Δ*ppa2*	2,2	Δ*mdm33*
Δ*mdm33* Δ*erg5*	2,21	Δ*mdm33*
Δ*mdm33* Δ*apq12*	2,23	Δ*mdm33*
Δ*mdm33* Δ*scs2*	2,23	Δ*mdm33*
Δ*mdm33* Δ*cbf1*	2,24	Δ*mdm33*
Δ*mdm33* Δ*ted1*	2,24	Δ*mdm33*
Δ*mdm33* Δ*bre5*	2,39	Δ*mdm33*
Δ*mdm33* Δ*sac1*	2,46	Δ*mdm33*
Δ*mdm33* Δ*sro7*	2,53	Δ*mdm33*
Δ*mdm33* Δ*bst1*	2,61	Δ*mdm33*
Δ*mdm33* Δ*snf4*	2,74	Δ*mdm33*
Δ*mdm33* Δ*arv1*	2,88	Δ*mdm33*
Δ*mdm33* Δ*ice2*	3,03	Δ*mdm33*
Δ*mdm33* Δ*bem1*	3,08	Δ*mdm33*
Δ*mdm33* Δ*cho2*	3,12	Δ*mdm33*
Δ*mdm33* Δ*mrc1*	3,13	Δ*mdm33*
Δ*mdm33* Δ*sec66*	3,16	Δ*mdm33*
Δ*mdm33* Δ*rps4a*	3,17	Δ*mdm33*
Δ*mdm33* Δ*opi3*	3,52	Δ*mdm33*
Δ*mdm33* Δ*ilm1*	3,63	Δ*mdm33*
Δ*mdm33* Δ*mdm12*	3,71	Δ***mdm12***
Δ*mdm33* Δ*gem1*	3,73	Δ***gem1***
Δ*mdm33* Δ*mdm35*	3,76	Δ*mdm33*
Δ*mdm33* Δ*mdm34*	4,12	Δ***mdm34***
Δ*mdm33* Δ*hof1*	4,26	Δ*mdm33*
Δ*mdm33* Δ*phb2*	4,91	**intermediate**
Δ*mdm33* Δ*psd1*	5,23	Δ*mdm33*
Δ*mdm33* Δ*phb1*	5,51	**intermediate**
Δ*mdm33* Δ*fmp30*	5,89	**intermediate**

75 double mutants were generated using the SGA technology, cells expressing mitochondrial matrix targeted GFP (mtGFP) were grown to logarithmic growth phase in YPD, and mitochondrial morphology was analyzed by fluorescence microscopy. The table includes the scores for the genetic interaction between both deleted genes according to the MITO-MAP^8^. Mitochondrial phenotypes in which the characteristic Δ*mdm33* morphology was not prevalent are highlighted in bold type.
