# Supplementary material for: Interaction of MDM33 with mitochondrial inner membrane homeostasis pathways in yeast
Source: Sci Rep. 2015 Dec 16;5:18344. doi: 10.1038/srep18344 (PMC4680886; doi:10.1038/srep18344)
Supplement: Supplementary Information [file srep18344-s1.pdf]

SUPPLEMENTARY MATERIALS

Interaction of *MDM33* with mitochondrial inner membrane homeostasis pathways in yeast

Till Klecker[1], Megan Wemmer[2], Mathias Haag[3], Alfons Weig[4], Stefan Böckler[1], Thomas Langer[3], Jodi Nunnari[2], and Benedikt Westermann[1,*]

[1]Universität Bayreuth, Zellbiologie, 95440 Bayreuth, Germany

[2]University of California Davis, Department of Molecular and Cellular Biology, Davis, CA 95616, USA

[3]Universität zu Köln, Institut für Genetik, 50931 Köln, Germany

[4]Universität Bayreuth, DNA Analytik, 95440 Bayreuth, Germany

*benedikt.westermann@uni-bayreuth.de

Content:

Supplementary figures 1-4

Legend to supplementary tables 1 and 2

Supplementary methods

Supplementary references

Supplementary figures

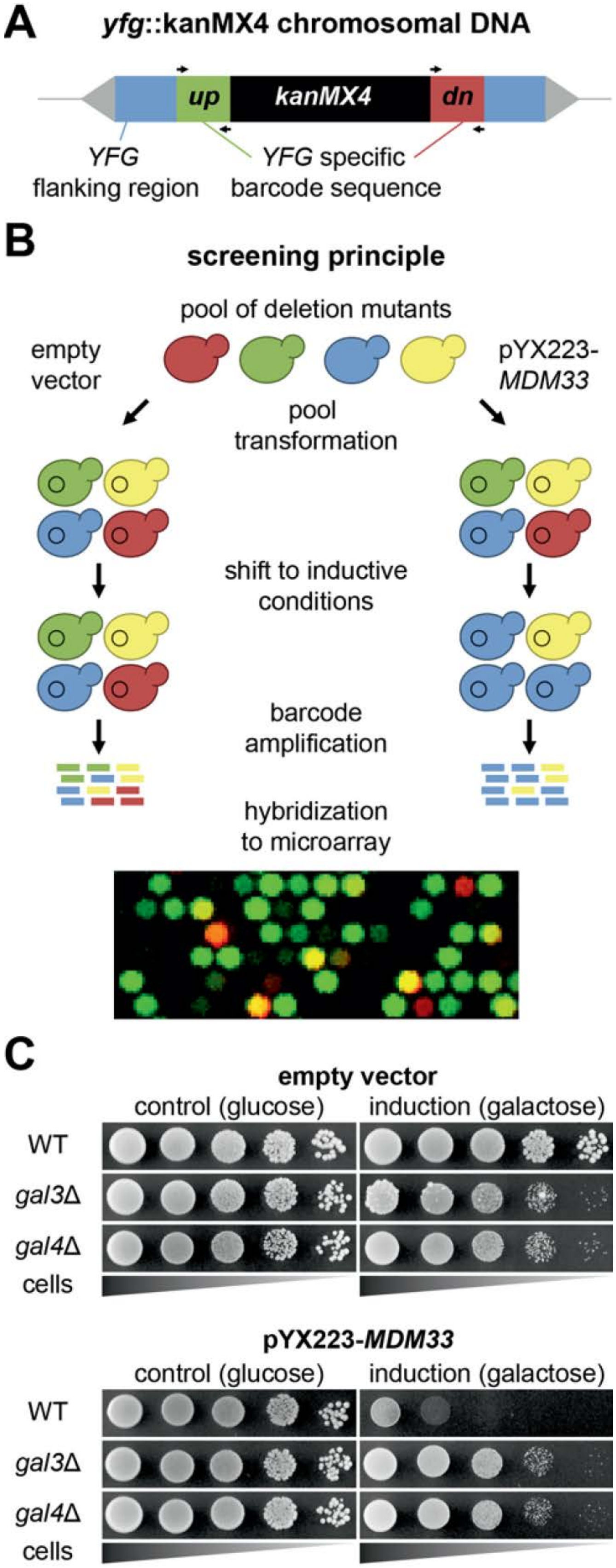

Figure S1. Screen for genetic interaction partners of *MDM33*. (A) Schematic representation of a bar-coded deletion allele in strains of the yeast deletion collection[1]. (B) Outline of the microarray-based genome wide suppressor screen. pYX223-*MDM33* was used for overexpression of *MDM33* from the *GAL1/10* promoter. (C) Strains were transformed with a multicopy plasmid for overexpression of *MDM33* from the inducible *GAL1/10* promoter (pYX223-*MDM33*) or the respective empty vector. 10-fold serial dilutions were spotted on synthetic complete medium containing glucose or galactose as carbon source and incubated at 30°C for 2 (glucose) or 4 days (galactose).

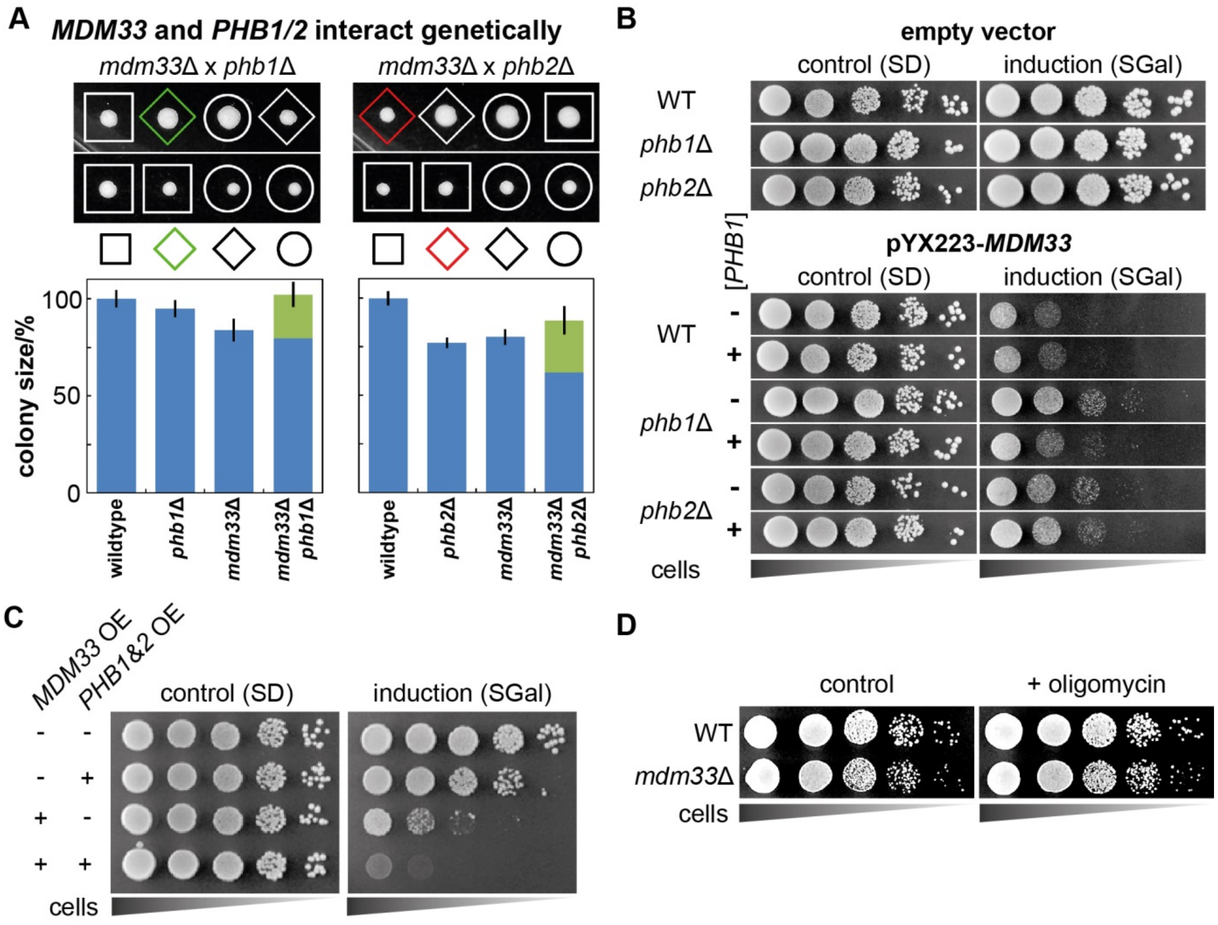

Figure S2. *PHB1* and *PHB2* genetically interact with *MDM33*. (A) Double mutants of Δ*phb1/2* and Δ*mdm33* were generated by tetrad dissection. The growth of more than 150 spores was scored for each cross and set in relation to the mean growth of the wild type spores. Shown are representative tetrads and the mean colony size in percent of wild type. Error bars indicate standard error. Green bars indicate the difference of the observed colony size and the size that would be expected if the genes would not interact. (B) Strains harboring a multicopy plasmid for overexpression of *MDM33* from the inducible *GAL1/10* promoter (pYX223-*MDM33*) or the respective empty vector were transformed with a low copy plasmid for expression of *PHB1*. 10-fold serial dilutions were spotted on synthetic complete medium containing glucose (repression of the *GAL* promoter) or galactose (induction of the *GAL* promoter) as carbon source and incubated at 30°C for 2-4 days. (C) 10-fold serial dilutions of strains overexpressing *MDM33* from plasmid pYX223-*MDM33* (*MDM33* OE) and/or both prohibitin genes under control of the *GAL1/10* promoter (*PHB1&2* OE) were spotted on synthetic complete medium containing glucose (control) or galactose (induction) as carbon source and incubated at 30°C for 2-4 days. (D) 10-fold serial dilutions of wild type and Δ*mdm33* cells were spotted on glucose-containing medium (YPD) without (control) or with the addition of 5 µg/ml oligomycin and incubated at 30°C for 2 days.

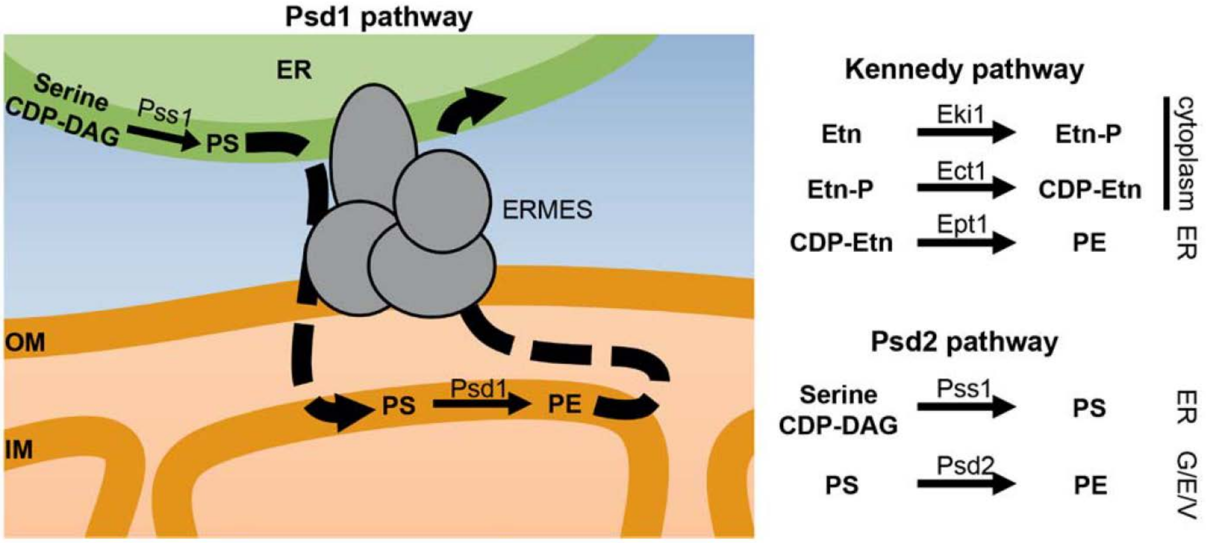

Figure S3. Phosphatidylethanolamine biosynthesis pathways in yeast. Dashed lines indicate transport, solid lines indicate enzymatic reactions. OM, mitochondrial outer membrane; IM, mitochondrial inner membrane; G/E/V: Golgi / endosome / vacuole; ERMES, ER-mitochondria encounter structure; PS, phosphatidylserine; PE, phosphatidylethanolamine; Etn, ethanolamine; Etn-P, phosphorylethanolamine; CDP-Etn, CDP-ethanolamine; CDP-DAG, CDP-diacylglycerol.

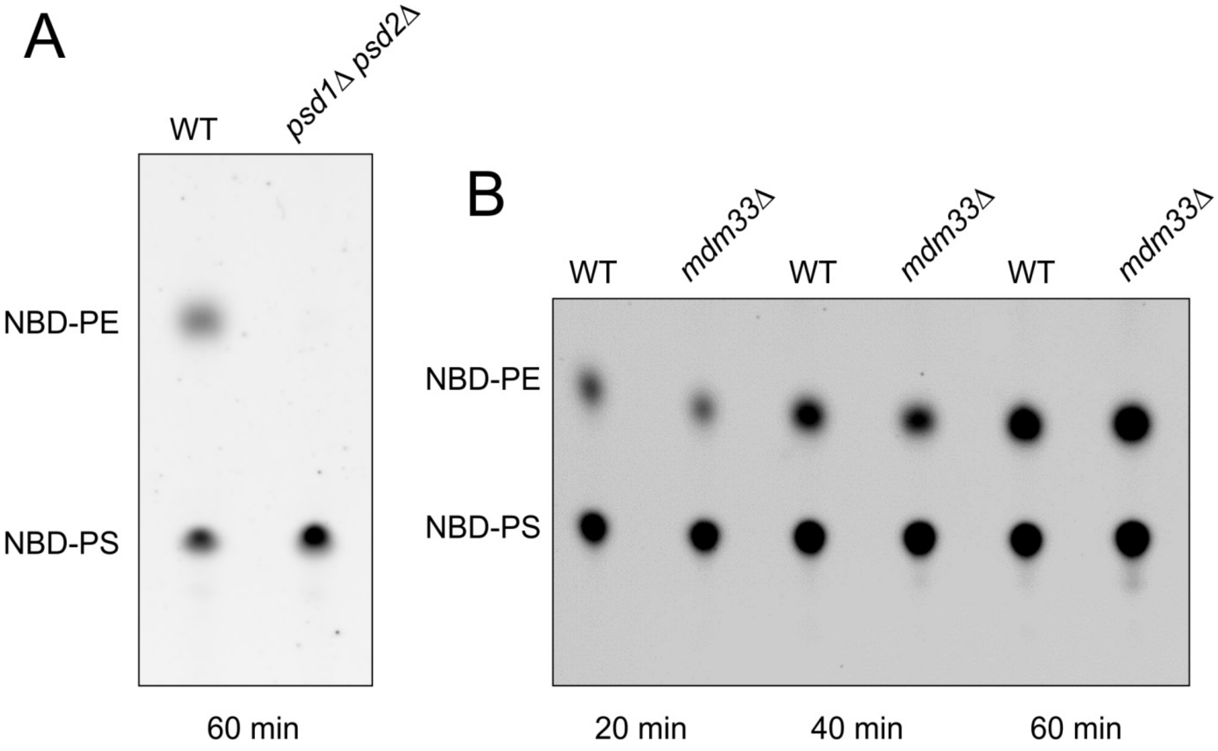

Figure S4. PS to PE conversion is unaffected in Δ*mdm33* mitochondria. (A) Mitochondria were isolated from a Δ*psd1* Δ*psd2* strain and incubated with liposomes containing NBD-PS. Total lipids were isolated and separated by TLC. Shown is the NBD-fluorescence. (B) Wild type and Δ*mdm33* mitochondria were incubated with liposomes containing NBD-PS for the indicated time periods and analyzed as above.

Table S1. MITO-MAP interaction scores of Δ*mdm33*. The table lists genetic interaction scores of the Δ*mdm33* deletion (alternative gene names *SHE9* or *YDR393w*) extracted from the MITO-MAP[2]. Interacting genes are ordered according to their interaction scores in ascending order. Highly significant negative interactions (score less than -3) are labelled in red, highly significant positive interactions (score more than 3) are labelled in green.

Table S1 is available as a separate Excel spreadsheet.

Table S2. Scores from the microarray-based genome-wide screen for suppressors of *MDM33* overexpression induced toxicity. A pool containing the 4,987 strains of the *MAT*α haploid non-essential yeast deletion library was transformed with pYX223-*MDM33*, plated on synthetic complete medium containing glucose or galactose as carbon source, and strain abundance was quantified by microarray hybridization. Normalized and background corrected microarray fluorescence signal values for the barcodes of each deletion strain are indicated for both conditions. The table also includes scores for the genetic interaction between the indicated gene and *MDM33* and their respective interactome similarity. Latter scores were taken from the MITO-MAP[2]. Red color indicates negative genetic interactions, green color indicates positive genetic interactions with a threshold of -3 and +3, respectively.

Table S2 is available as a separate Excel spreadsheet.

Supplementary methods

Plasmids and cloning procedures

Standard procedures were used for cloning and amplification of plasmids. PCR was performed using Pfu polymerase (Fermentas, St. Leon-Rot, Germany) or the GoTaq polymerase (Promega, Madison, WI) according to the manufacturer's instructions. Plasmids pYX142-mtGFP[3], pVT100U-mtGFP[3], pYX142-mtERFP[4], pYX223-MDM33[5], pHS20[6], pTT46(Phb1)[7], pESC-SCO2[8], pESC-YHM2[8], and pRS316-MMM1-ERFP[9] were described previously. To obtain pYX223-mtBFP, the BFP fused to the Su9 mitochondrial presequence was subcloned from pYES-mtBFP[3] into the *Hin*dIII and *Xho*I sites of pYX223. The FLAG-tagged version of *MDM33* was constructed by PCR-amplification-based fusion of the FLAG tag to the fragment encoding the mature part of *MDM33* using the oligonucleotides 5'-ATA TAT GGA TCC GAT TAT AAA GAT GAT GAC GAT AAG CTA CAG AAC GGT GAT ACT CC-3' and 5'-AAT TTT CTC GAG TTT TAA CGA TAT TCT TGC GC-3' and genomic DNA as template. This fragment was then cloned into the *Bam*HI and *Xho*I sites of pMM112[5], yielding pRS316-FLAG-MDM33. Plasmid pYX223-FLAG-MDM33 was created by PCR-amplification of the fragment containing the import sequence, the FLAG tag and the mature part of *MDM33* using pRS316-FLAG-MDM33 as template and oligonucleotides 5'-TAT AAA GCT TAT GTT GAG ATA CTA TGG GGC GAC-3' and 5'-AAT TTT CTC GAG TTT TAA CGA TAT TCT TGC GC-3' and cloning into the *Hind*III and *Xho*I sites of pYX223. Simultaneous overexpression of *PHB1* and *PHB2* was achieved using the *GAL1/10* promoter-containing pESC-URA vector[7]. Plasmid pTT21 for expression of untagged Phb1 and Phb2 was a kind gift from Takashi Tatsuta (Universität zu Köln, Germany).

Antibodies

A monoclonal anti FLAG antibody (FLAG-M2; catalogue F3165) was purchased from Sigma-Aldrich (St. Louis, MO, USA). Polyclonal anti hexokinase (Hxk1) antiserum (catalogue 100-4159) was purchased from Biotrend Biochemikalien GmbH (Köln, Germany). Polyclonal anti Mgm1 antiserum was a kind gift from Andreas Reichert (Universität Düsseldorf, Germany), polyclonal anti Psd1 antiserum was a kind gift from Steven M. Claypool (Johns Hopkins School of Medicine, Baltimore, MD), and polyclonal anti Tom40 antiserum was a kind gift from Doron Rapaport (Universität Tübingen, Germany).

Microarray design and hybridization

The *S. cerevisiae* TAG microarray design is based on the Saccharomyces Genome Deletion Project[1]. The 20mer UpTAG and DownTAG sequences were taken from the *MAT*α mating type strains. Reinvestigations of the yeast knockout strain collection by deep sequence analysis of the TAG sequences revealed yeast deletion strains that contained mutated TAG

barcodes in comparison to the originally designed barcodes[10, 11]. We included the originally designed barcodes as well as the combined mutated barcodes of the two re-sequencing projects in our microarray design. If the mutated sequence contained deletions of one or more nucleotides, the mutated sequence was filled up with Ts to a 20mer sequence. In addition, a limited set of 500 mismatches of UpTAG sequences were designed by exchange of the eleventh nucleotide (A↔T, G↔C). Furthermore, the primer sequences used for labeling the TAG barcodes were also included in the microarray design. All TAG sequences were uploaded to the Agilent eArray website and extended to a final length of 60 nucleotides with Agilent's linker sequences. Probes were randomly distributed across the array using the 8x15k format and printed slides were ordered directly from Agilent Technologies (Waldbronn, Germany).

Genomic DNA from yeast samples was extracted using the NucleoMag 96 Plant DNA extraction kit (Machery-Nagel, Dueren, Germany) on a KingFisher magnetic particle processor (Thermo Scientific, Langenselbold, Germany). Genomic DNA was precipitated with ethanol and suspended in TE buffer. TAG barcode sequences were labeled by asymmetric PCR (20 µl) using 200 ng genomic DNA as described[12]. After PCR labeling, 3.6 µl blocking oligonucleotides U1c and U2c (50 µM each) were added to 1.5 µl of the upTAG PCR labeling reaction, heated to 100°C for 1 min, and kept at room temperature for at least 20 min; downTAG samples were treated in the same way with downTAG blocking oligonucleotides D1c and D2c. A hybridization solution was prepared by combining Cy3- and a Cy5-blocked TAG samples (each 4.5 µl), 9 µl nuclease-free water, 4.5 µl 10X Blocking Agent and 22.5 µl 2X Hi-RPM Buffer (Agilent Technologies). The hybridization mix was added to the Agilent 8x15k arrays and hybridized at 50°C for ca. 16 h. Microarrays were washed using Oligo aCGH Wash Buffer 1 and 2 (Agilent Technologies) as recommended by the manufacturer; decomposition of cyanine dyes was protected by incubating the slides in a Stabilization and Drying Solution (Agilent Technologies).

Dry microarray slides were scanned in a FLA8000 slide scanner (Fujifilm) at 5 µm pixel resolution in confocal mode. Microarray images were analyzed using the ArrayVision software v8.0, rev4.0 (GE Life Sciences); spot intensities were calculated as background-corrected median-based trimmed mean densities. Spot values of each microarray experiment were scaled to a total array intensity of 147,680,000 units to facilitate comparison of independent experiments (total intensity normalization)[13].

Immunoprecipitation and LC MS/MS analysis

Immunoprecipitation was performed basically as described previously[2] with the following adjustments. 500 ODUs of GFP-tagged strains or an untagged W303 control were lysed in IPLB (20 mM Hepes, pH 7.4, 150 mM KOAc, 2 mM $Mg(Ac)_2$, 1 mM EGTA, and 0.6 M

sorbitol) with 1X protease inhibitor cocktail set I (CalBiochem) on ice for 10 min and cleared. Lysates were crosslinked by addition of 1 mM DSP (Thermo Scientific) and allowed to incubate on ice for 30 min. Crosslinking was quenched with the addition of 100 mM Tris pH 7.4. Membranes were then solubilized with 1% digitonin on ice for 30 minutes, and subsequently cleared at 12,000x g for 10 min at 4˚C. The supernatant was incubated with 50 µl antiGFP microbeads (Miltenyi Biotec Inc.) on ice for 30 min. The beads were isolated using Miltenyi µ columns and a µMACS separator (Miltenyi Biotec Inc.), washed three times with IPLB with 0.1% digitonin and protease inhibitors, and washed twice with IPLB containing no digitonin or protease inhibitors. Onbead trypsin digestion was performed, beads were incubated for 30 min at room temperature in 25 µl elution buffer I (2 M urea; 50 mM Tris, pH 7.5, 1 mM DTT, and 5 µg/ml trypsin). This was followed by two 50 µl applications of elution buffer II (2 M urea; 50 mM Tris, pH 7.5, and 5 mM chloroacetamide). Elutions were collected and digestion was allowed to continue at room temperature overnight. Reactions were stopped with 1 µl trifluoroacetic acid (TFA). Peptides samples were submitted to the Genome Center Proteomics Core at the University of California, Davis, for mass spectrometry (LC MS/MS) based protein identification. Urea from the peptide samples was removed using desalting tips (Aspire RP30; Thermo Fisher Scientific). The tips were prepared by pipetting 60% acetonitrile up and down 20 times and then equilibrated with 0.1% TFA by pipetting up and down 15 times. The peptide sample was pulled through the tip 15 times before being washed with 0.1% TFA. The peptides were eluted from the tips using 100 µl of 60% acetonitrile and dried via vacuum centrifugation. Protein identification was performed using a Paradigm HPLC and CTC Pal autosampler (both from Bruker) paired to either a LTQ ion trap mass spectrometer (Thermo Fisher Scientific) or Thermo-Finnigan LTQFT ultra ion trap mass spectrometer (Thermo Fisher Scientific) through an ADVANCE Plug and Play Nano Spray Source (Bruker). Peptides were desalted onto a nanotrap (Zorbax 300SBC18; Agilent Technologies), then eluted from the trap and separated by a 200 mm × 15 cm Magic C18 AQ column (Bruker) at a flow rate of 2 µl/min. Peptides were eluted using a 60 min gradient of 2-80% buffer B (buffer A, 0.1% formic acid; buffer B, 95% aceto nitrile/0.1% formic acid). The elution gradient was set at 2-35% buffer B for 30 min, increased from 35-80% buffer B for 2 min, and held at 80% buffer B for 1 min. The gradient then decreased from 80-2% buffer B over 2 min and equilibrated for 25 min. The top 10 ions in each survey scan were subjected to automatic low energy collision induced dissociation. Tandem mass spectra were extracted by BioWorks version 3.3. Mass charge state deconvolution and deisotoping were not performed. All MS/MS samples were analyzed using X! Tandem. The raw data was analyzed with X! Tandem using the UniProt *Saccharomyces cerevisiae* database appended with the cRAP database, which includes a compilation of common laboratory contaminants, and both forward and reverse sequences were utilized in the data analysis. Trypsin was set as the

cleaving enzyme in the X! Tandem search parameters. X! Tandem was searched with a fragment ion mass tolerance of 0.4 D and a parent ion tolerance of 1.8 D. Iodoacetamide derivative of cysteine was specified in X! Tandem as a fixed modification. Deamidation of asparagine and glutamine, oxidation of methionine and tryptophan, sulfone of methionine, tryptophan oxidation to formylkyn urenine of tryptophan, and acetylation of the N terminus were specified in X! Tandem as variable modifications. Scaffold (version Scaffold_2_02_033_00_07; Proteome Software Inc.) was used to validate MS/MS based peptide and protein identifications. Proteins with a t test P value ≤ 0.05 comparing unique peptides obtained from mass spec of tagged strain to untagged control were further investigated.

Supplementary references

1. Giaever, G. *et al.* Functional profiling of the *Saccharomyces cerevisiae* genome. *Nature* **418**, 387-391 (2002).
2. Hoppins, S. *et al.* A mitochondrial-focused genetic interaction map reveals a scaffold-like complex required for inner membrane organization in mitochondria. *J. Cell Biol.* **195**, 323-340 (2011).
3. Westermann, B. & Neupert, W. Mitochondria-targeted green fluorescent proteins: convenient tools for the study of organelle biogenesis in *Saccharomyces cerevisiae*. *Yeast* **16**, 1421-1427 (2000).
4. Scholz, D., Förtsch, J., Böckler, S., Klecker, T. & Westermann, B. Analyzing membrane dynamics with live cell fluorescence microscopy with a focus on yeast mitochondria. *Meth. Mol. Biol.* **1033**, 275-283 (2012).
5. Messerschmitt, M. *et al.* The inner membrane protein Mdm33 controls mitochondrial morphology in yeast. *J. Cell Biol.* **160**, 553-564 (2003).
6. Sesaki, H. & Jensen, R.E. Division versus fusion: Dnm1p and Fzo1p antagonistically regulate mitochondrial shape. *J. Cell Biol.* **147**, 699-706 (1999).
7. Tatsuta, T., Model, K. & Langer, T. Formation of membrane-bound ring complexes by prohibitins in mitochondria. *Mol. Biol. Cell* **16**, 248-259 (2005).
8. Zhu, H. *et al.* Global analysis of protein activities using proteome chips. *Science* **293**, 2101-2105 (2001).
9. Böckler, S. & Westermann, B. Mitochondrial ER contacts are crucial for mitophagy in yeast. *Dev. Cell* **28**, 450-458 (2014).
10. Eason, R.G. *et al.* Characterization of synthetic DNA bar codes in *Saccharomyces cerevisiae* gene-deletion strains. *Proc. Natl. Acad. Sci. USA* **101**, 11046-11051 (2004).
11. Smith, A.M. *et al.* Quantitative phenotyping via deep barcode sequencing. *Genome Res.* **19**, 1836-1842 (2009).
12. Pan, X. *et al.* dSLAM analysis of genome-wide genetic interactions in *Saccharomyces cerevisiae*. *Methods* **41**, 206-221 (2007).
13. Quackenbush, J. Microarray data normalization and transformation. *Nat. Genet.* **32 Suppl**, 496-501 (2002).
